# Supplementary figures and images for: Natural image statistics for mouse vision
Source: PLoS One. 2022 Jan 20;17(1):e0262763. doi: 10.1371/journal.pone.0262763 (PMC8775586; doi:10.1371/journal.pone.0262763)

**A** lower visual field images  
(N=117)

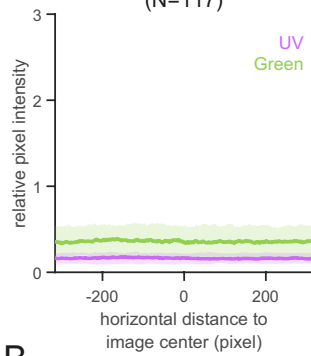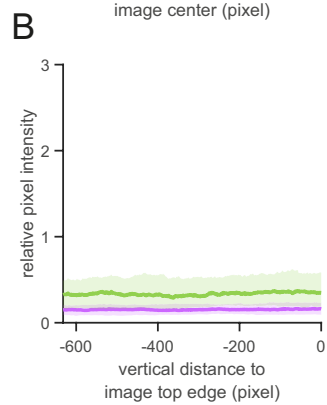

**C** horizontal visual field images  
(N=15)

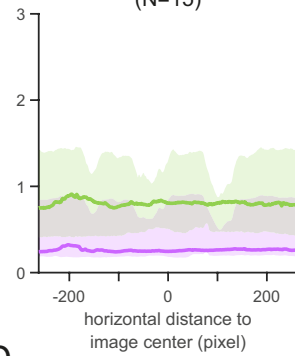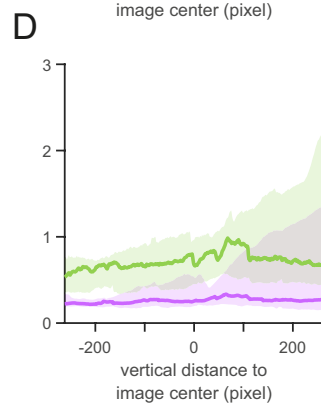

**E** upper visual field images  
(N=100)

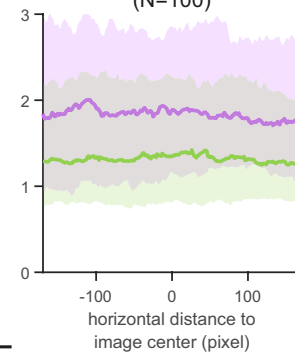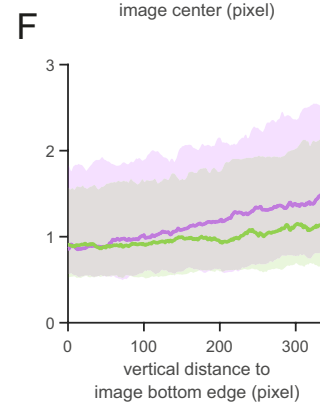

Supplement: S1 Fig — Relative pixel intensities (median ± interquartile range; UV and green channels in violet and green, respectively) were computed along horizontal (A,C,E) and vertical (B,D,F) axes for three different image categories based on the camera angle: Lower (A,B; N = 117), horizontal (C,D; N = 15), and upper (E,F; N = 100) visual field images. Pixel intensity did not change much horizontally but was generally lower in the lower field images (A,B) than in the upper field images (E,F). Discontinuity between the top edge of the lower field images (B, x-axis value of 0) and the bottom edge of the upper field images (F, x-axis value of 0) supports a good separation of the two image categories. (PDF) [file pone.0262763.s001.pdf]

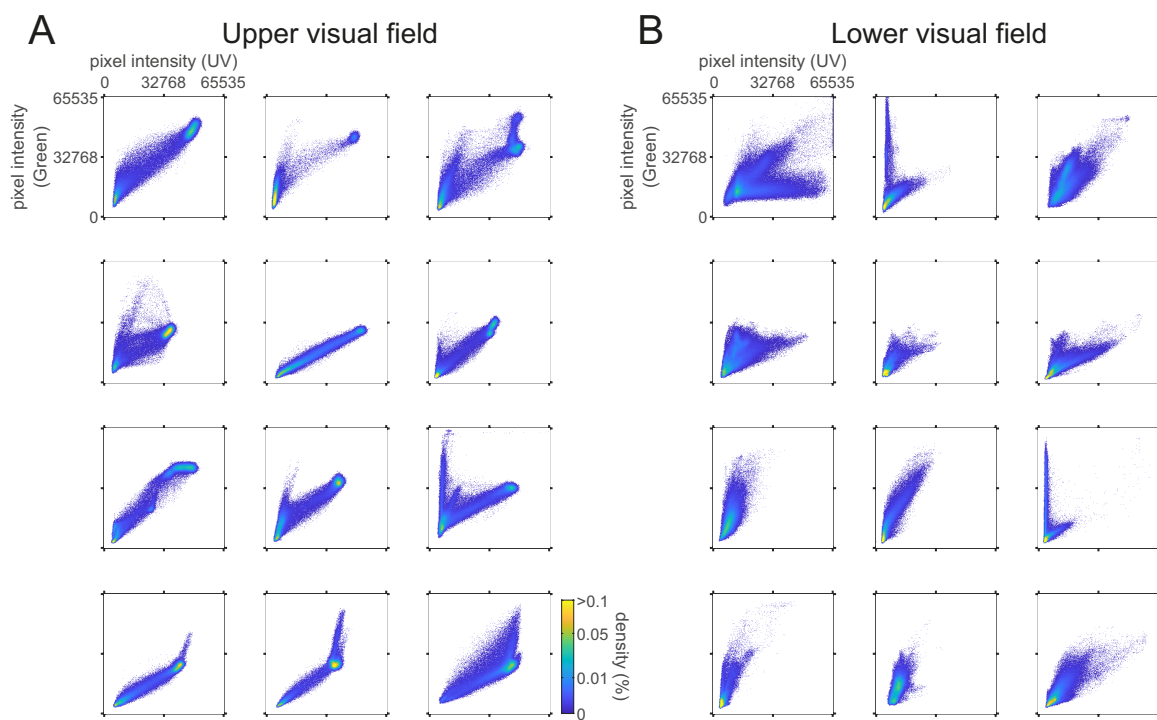

Supplement: S2 Fig — Each scatter plot shows the distribution of the UV-Green pixel values from the corresponding image shown in Fig 2 (A, upper visual field images; B, lower visual field images). Virtually all pixels were within the dynamic range of the camera sensor (Sony, IMX174 CMOS; 12-bit depth saved in a 16-bit format). (PDF) [file pone.0262763.s002.pdf]

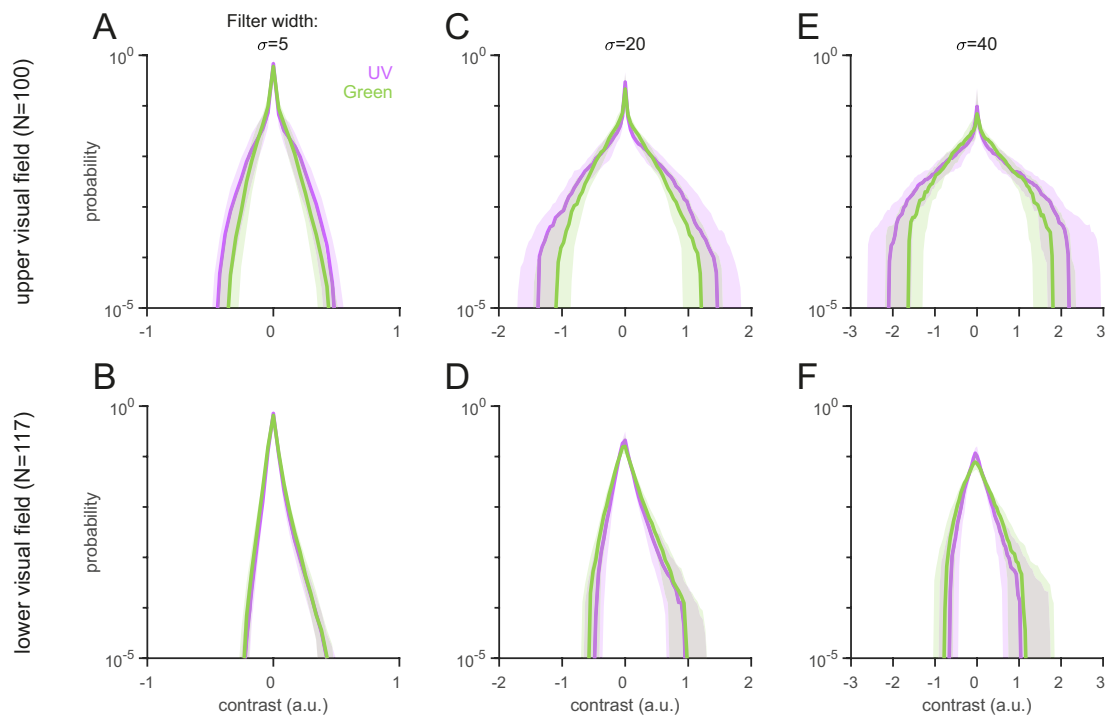

Supplement: S3 Fig — Local contrast distributions computed with different Laplacian-of-Gaussian filter sizes (A,B, σ = 5; C,D, σ = 20; E,F, σ = 40; Eq (2)) are shown in the same format as Fig 3C and 3D (σ = 10). The upper visual field images (A,C,D) generally showed higher contrast than the lower visual field images (B,D,F), especially for the UV channel (violet). The filter size (0.18–1.44 degrees) used in this study is smaller than the receptive field size of mouse retinal ganglion cells (3–13 degrees) [72,73]. Given the scale invariance [2,21], however, we expect that our analysis results should hold for larger filters as well [22]. (PDF) [file pone.0262763.s003.pdf]

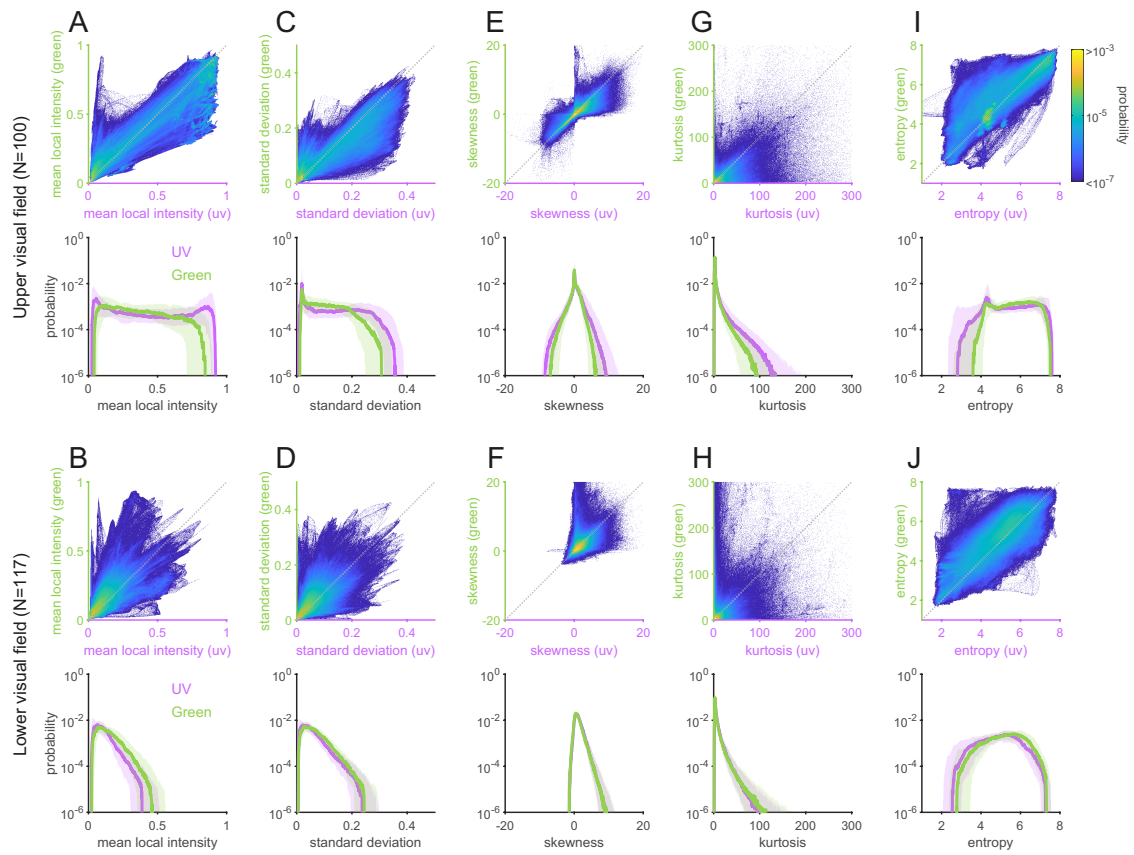

Supplement: S4 Fig — The first- to the fourth-order image statistics (mean, A, B; standard deviation, C, D; skewness, E, F; kurtosis, G, H) as well as entropy (I, J) were computed for local images patches (0.36 degrees; UV, violet; Green, green). Joint (top) and marginal (bottom) probability distributions were then generated for the upper (A, C, E, G, I) and lower (B, D, F, H, J) visual field images. (PDF) [file pone.0262763.s004.pdf]
