# Supplementary material for: Natural image statistics for mouse vision
Source: PLoS One. 2022 Jan 20;17(1):e0262763. doi: 10.1371/journal.pone.0262763 (PMC8775586; doi:10.1371/journal.pone.0262763)
Supplement: Attachment — Submitted filename: Response_to_reviewers.docx [file pone.0262763.s005.docx]

We thank the reviewers for their constructive comments. We have addressed all the concerns (in blue). Below are point-by-point replies (in black).

Reviewer #1: The article by Abballe and Asari entitled " Natural Image Statistics for Mouse Vision" demonstrated a camera system for taking photos with dichromatic spectra: UV and green, corresponding to sensitivities of S-opsin (UV) and M-opsin (green) for the cone photoreceptors of mouse retina. Using this camera system, they acquired photos of natural scenes without artificial objects. The authors divided the image data into two main groups (upper and lower vision field). By analyzing 1) the pixel-by-pixel intensity (first order statistics) and 2) luminance contrast, power spectrum and spatial autocorrelation (second order statistics) in the two chromatic channels, the authors found distinct properties between two channels: higher local contrast and spatial autocorrelation in UV in the upper visual field, while they were lower in the lower visual field.

Comments/questions/suggestions:

1) This camera system used a 50mm lens to capture the nature sense. It would be good to know what the field of view (FOV) of the whole camera system is. Did they ever change the image resolution? In the mouse retina, the spatial resolution is determined by the receptive field (RF) size of the ganglion cells. The visual angle of different ganglion cell types varies from 3 to 13 degree. Thus, the local contrast may change with the RF sizes. Since the authors calculated the local contrast using a fixed area XY in [-30 pixels, +30 pixels], the local contrast may only represent the contrast for certain ganglion cell types.

To maximize image quality, we chose a large field of depth (f = 22) and a small field of view (FOV): 11.3 degrees horizontally and 7.3 degrees vertically. Thus, the pixel size of our acquired images (1920-by-1200 pixels; 12 bit depth) is 0.006 degrees/pixel. These original images were preprocessed for image registration and vignetting correction, cropped into 341-1766 pixels (2.0-10.6 degrees) horizontally and 341-1120 pixels (2.0-6.7 degrees) vertically, and saved in the 16-bit grayscale Portable Network Graphic (PNG) format. We never changed the image resolution. See the **revised** **Methods section** (**lines 73-75 and 134-137**) and the scale bar in the **revised Fig. 2**.

Natural image statistics are scale invariant (Simoncelli and Olshausen, 2001; Hyvärinen et al., 2009). This is why we chose a rather arbitrary size of the filter width (sigma of the LoG filter = 10 pixels = 0.06 degrees) to calculate the local contrast distribution. Indeed, we confirmed that the contrast distributions have a similar shape regardless of the filter size (**S3 Fig** in the revised manuscript) where we tested filters with σ = 5-40 pixels (= 0.03-0.24 degrees), corresponding to the full filter size (6 σ) of 0.18-1.44 degrees. We could not examine a larger filter size due to limited image sizes. Thus, the filter size used in this study is smaller than the receptive field size of mouse retinal ganglion cells (3-13 degrees). Nevertheless, given the scale invariance, we expect that our analysis results with small filters should hold for larger filters as well. Indeed, we obtained similar results (higher contrast in UV than in green for upper field images) as in Qiu et al (2021) where larger filter sizes (2-14 degrees) were used. We clarified these points in the **revised Methods section (lines 163-165)** and **S3 Fig legend (lines 649-651)**.

2) In Figure3 A&B, the authors plotted the normalized intensity across the data sets. I am wondering if the authors did the calibration for the camera system. The camera may be underexposure for dark scenes and overexposure for bright scenes, which depends on camera settings. Both underexposure and overexposure can result in missing luminance information. There are quite pixels with 0 value in the figure, indicating that images tended to be underexposure. However, these data are normalized between 0-1. It is difficult to check their original values. Moreover, the authors used two different ND filters in green channel to increasing the dynamic range. In my view, this design also changed the sensitive of the green part even though the camera settings remained same. So, two images taken with different ND filters cannot be compared for contrast. Thus, the promising solution is to calibrate the system, relating the intensity of each pixel to the power of the light at defined wavelength. Another advantage of calibration can avoid gamma correction of any graphic-related system.

We chose the Sony IMX174 CMOS sensor because it shows a linear relationship between the pixel intensity values (12 bit depth; saved in 16-bit grayscale) and light intensity. See page 9 of the following references:

Jähne & Herrmann (2015) Zenodo DOI: 10.5281/zenodo.33040

Jähne & Herrmann (2015) Zenodo DOI: 10.5281/zenodo.33041

We have also confirmed the linearity after vignetting correction by taking uniform images (of a clear sky on a sunny day) at different exposure times (**revised Fig. 1A, inset**).

Before taking each image, we checked the intensity histogram and chose a proper exposure value (and an ND filter for the green channel; OD 1.0, 1.3, 1.5., 1.8, or 2.0) so that virtually all the pixels were within the dynamic range of the sensor. All the images were taken without any image corrections (gamma = 1). Thus, our image data sets (232 images) have no underexposed pixels and only a negligible number of overexposed pixels (0.0011% of pixels in 2 UV images and 0.0007% in 6 green images). We clarified the point in **lines 95-102 of the revised manuscript**. We also made the following revisions to clarify that virtually no information was lost during the image acquisition:

1) **S2. Fig in the revised manuscript** shows scatter plots of UV-green pixel intensity values before normalization for the representative images shown in Fig. 2. Note that those images in Fig. 2 appear dark because they were not gamma corrected.
2) **Revised Fig. 3A,B** shows the probability distributions of the normalized pixel values (median +/- interquartile range) using a smaller bin size (0.001 instead of 0.01) for a better representation.

Following the Weber-Fechner law, contrast is essentially a ratio of “luminance difference / overall luminance level” (though there are several different definitions of contrast). Thus, contrast is not affected by the absolute power of light. Because neutral density (ND) filters reduce the luminance by the same amount uniformly across pixels and spectra, they do not affect contrast as long as all the pixels are within the dynamic range of the sensor. Note that, in this study, we first normalized the dynamic range (i.e., maximum contrast) of each image, and then used a Laplacian-of-Gaussian filter (Eq.(2)) to compute local luminance difference in each image patch (as in a classical “center-surround” receptive field of retinal ganglion cells).

We also measured the root mean square (RMS) contrast (Eq.(4)) of our image data sets, and found the same results (higher contrast in UV for upper visual field images; **new Fig 4A,B in the revised manuscript**). This is also consistent with Qiu et al. (2021).

3) In the abstract, “… natural image statistics of UV light have never been extensively assessed beyond spectral analysis. …”. However, in the discussion part (line: 339), the authors mentioned a very similar camera system with related statistics (now published as Qiu et al., 2021). Could the authors compare the difference between these two camera systems?

We identified certain hardware differences between Qiu et al. (2021) and our study:

1) The biggest difference is the field of view (FOV). Our system has a small FOV to minimize image distortion for properly analysing higher-order image statistics. It also allows us to properly adjust exposure times to fully capture the dynamic range of individual scenes. In contrast, Qui et al. (2021) used a fish-eye lens to capture hemispherical images. These images inevitably had greater distortion and were susceptible to overexposure on the upper side (sky) while underexposure on the lower side (ground); moreover, lens noise and vignetting were not corrected in their image data sets.
2) Our sensor (Sony IMX174) has a larger bit depth (12 bit versus 8 bit), higher pixel resolution (1920-by-1200 versus 720-by-540), and the linearity (as described above). It also has high quantum efficiency (~30% at 365 nm; ~75% at 510 nm), low dark noise (7 e-) and high dynamic range (73 dB) with a global shutter. See the datasheet here:

<https://www.flir.com/globalassets/support/iis/whitepaper/sony-pregius-global-shutter-cmos-imaging-performance.pdf>

3) Our system has fewer optical elements to ensure transmission of near ultraviolet (UV) light. The spectral sensitivity of our system (i.e., convolution of the measured transmission spectra of the entire optics and the spectral sensitivity of the sensor obtained from the abovementioned datasheet) was all within the UV range (<400 nm; Fig. 1B), with the peak sensitivity (368 nm) closer to that of the mouse UV vision (365 nm). Note that Qiu et al (2021) did not report the spectral sensitivity of their system (besides the transmission spectra of the dichroic and bandpass filters; e.g., 350-419 nm for the UV channel); and that they took images with different settings on the two cameras (higher gain for UV images).

We therefore think that our image datasets have better image quality and are more suitable for analyzing image statistics, especially for higher-order ones, such as spatial autocorrelation. To our knowledge, we are the first to provide such high-quality UV images comparable to the natural image datasets for human vision (Geisler and Perry, 2011; Hateren and Schaaf, 1998; Tkacik et al., 2011; Zhou et al., 2014). We clarified these points in the **revised Methods section (lines 71-81)**.

4) In the multi-spectral camera design part (line 84), this dichroic filter (Edmund Optics, 68-330) is just a silver mirror according to Edmund Optics. Could the authors double check the information of this item?

We apologize for the error in the method section. It should be “409nm, 25.2 x 35.6mm High Performance Fluorescence Dichroic Filter” (Edmund Optics, 34-725). We corrected the error in the revised manuscript (**line 60**).

5) Spatial autocorrelation is used to measure spatial dependence/similarity. In this study, this method calculated the autocorrelation within individual images, which depends on the content of each image. My hypothesis is that the results might be affected by the exposure settings for each scene in different chromatic channels. For example, right exposure of a tree can give more details and, hence, lower values, while underexposure can have higher correlation due to less details. Could the authors provide some data for different exposure settings from a same nature scene?

We agree that the presence of under- or over-exposed pixels will skew the results. As described above, however, we chose a proper exposure level for each scene so that virtually no pixel was under- or over-exposed (see examples in **S2 Fig** in the revised manuscript). Thus, the result should faithfully represent the auto-correlation structure of each image, regardless of the exposure or ND filter settings.

We also agree that the auto-correlation structure depends on the content of each image. This is the whole point of comparing the statistics of the upper- and lower-field images as they include different contents. Of course, some questions remain on how faithful our images are to what mice actually see in their natural habitats. In particular, caveats include that 1) the effects of the mouse eye optics were not considered in our image acquisition system; 2) no motion dynamics were considered; 3) images were taken under ample light during the day, while mice are nocturnal; and 4) our image datasets were still relatively small. It is a future challenge to address these questions, for example, by measuring the properties of the mouse eye optics and simulating images projected onto the mouse retina. We clarified these points in the **revised Discussion section (lines 413-421)**.

Reviewer #2: Abballe and Asari describe a UV/Green camera system that they have used to capture images of natural scenes in two spectral bands (a multi-spectral camera). They in turn analyse the natural image statistics in these two channels, and in two broad regions of the visual field (upper and lower). They find that the statistics differ across the spectral bands and regions, such that local contrast and spatial correlation are higher in the UV images taken in the upper visual field (compared with green), and lower in the UV images taken in the lower visual field (compared with green). In turn, they conclude that the mouse retina is not optimised to detect these differences, since the efficient coding hypothesis would predict optimal sampling with UV cones in the dorsal retina and green cones in the ventral (opposite to the known distribution). This work is novel at the time of submission, and will be valuable to the large community of researchers working on mouse visual physiology, in particular, a growing field researching the relevance of natural visual stimuli in visual coding.

I have a few questions that the authors should address:

I’d firstly like to know more about the camera and its calibration. In particular: what is its resolution (8 bit?); how linear is the detector, does it apply a gamma correction and if so, is it accounted for? Similarly, how sensitive is the camera to shorter (<400nm) wavelengths, and does this need to be accounted for in any way for the UV measurements?

The Sony IMX174 sensor has 12 bit depth with a linear response curve (**revised Fig. 1A, inset**):

Jähne & Herrmann (2015) Zenodo DOI: 10.5281/zenodo.33040

Jähne & Herrmann (2015) Zenodo DOI: 10.5281/zenodo.33041

No gamma correction was applied during image acquisition. The sensor has a quantum efficiency of ~30% at 365 nm, which was considered when the spectral sensitivity of our camera system was calculated (Fig. 1B). Specifically, it was computed by convolving the measured transmission spectra of the entire optics and the spectral sensitivity of the sensor (obtained from datasheet):

<https://www.flir.com/globalassets/support/iis/whitepaper/sony-pregius-global-shutter-cmos-imaging-performance.pdf>

We clarified this in the revised manuscript (**lines 76-80**). Please also see our response to the second comment by the first reviewer.

Most of the analysis compares between image pixels – some frame of reference would be useful to understand how these pixels relate to the mouse visual system. What is the camera’s field of view in degrees, and what would be the size of each pixel be in terms of visual angle? How does that relate to the receptive field size of neurons in the mouse’s eye?

Relating to this, how ‘large’ are the images in approximate visual space, and what area of the retina would this likely cover?

Our camera’s field of view (FOV) is 11.3 degrees horizontally and 7.3 degrees vertically, with a pixel size of 0.006 degrees/pixel (**lines 73-76** in the revised manuscript; see also the scale bar in the **revised Fig. 2**). The filter size we used to compute the contrast (0.18-1.44 degrees) is much smaller than the receptive field of mouse retinal neurons (3-13 degrees). However, given that natural image statistics are scale invariant (**S3 Fig in the revised manuscript**), we expect that the analysis results with small filter sizes in this study will hold for those with larger filter sizes. Please see also our response to the first comment by the first reviewer.

The mouse has roughly a hemispheric visual field for each eye, with the optical axis tilted ~64 degrees in azimuth towards front and ~22 degrees in elevation towards the sky (Sterratt et al 2013; Stabio et al., 2018). Unfortunately, we did not measure the exact angle of the camera when we took images. However, we categorized the images into three groups based on the camera angle (upper, horizontal, lower), and we excluded the “horizontal” images from the analysis to ensure that the “upper” and “lower” images correspond to those images falling into the ventral and dorsal parts of the mouse retina, respectively. Our images cover 2.0-10.6 degrees (341-1766 pixels) horizontally and 2.0-6.7 degrees (341-1120 pixels) vertically. **See S1 Fig and the revised Methods section (lines 134-136; 140-150).**

Are the images of upper and lower regions likely to occur within the same angle of view i.e. covering both dorsal and ventral parts of the retina, as the authors use for their analysis?

No, the upper and lower visual field images should not overlap each other. Indeed, the relative pixel intensity along the vertical axis has a clear discontinuity between the bottom edge of the upper field images and the top edge of the lower field images (**S1 Fig in the revised manuscript**). Thus, the upper and lower field images will likely be processed exclusively by the ventral and dorsal retina, respectively.

As mentioned above, we also took “horizontal” images that are likely projected onto around the equator of the mouse eye. These images were excluded from our analysis to ensure clear separation between the “upper” and “lower” image groups. We clarified this in the revised manuscript (**lines 140-150**).

A similar analysis has been published while this paper has been under review (Qiu et al, 2021, Current Biology). Their data are in line with Abballe and Asari, though they conclude that the mouse retina’s specialisations match (rather than oppose) the scene statistics - some discussion is probably needed here.

It is commonly assumed that the mouse retina has evolved to optimally process natural images in its natural habitats. It remains unclear, though, in what sense the mouse retina is optimal. Our study and Qiu et al (2021) made different conclusions on the relationship between the mouse retinal organization and natural image statistics because we had different perspectives. On the one hand, we took an information theoretic viewpoint and found that the coding efficiency by itself does not explain well the mouse retina’s specialisations given the natural image statistics. For example, we argue that, for equalizing the bandwidth, high contrast images in the upper visual field should be encoded with less sensitive photoreceptors (M-cones), while low contrast images in the lower visual field with more sensitive photoreceptors (S-cones). In contrast, Qiu et al (2021) argued from an ethological viewpoint that more sensitive S-cones are driven more strongly by high-contrast upper-field images and thus better suited to process biologically relevant information for survival, such as detecting aerial predators. These two arguments are compatible with each other, and it is a future challenge to clarify what exactly those ethological constraints are and how they drove species-specific evolution of the mouse retina. These are important questions, especially because many other small animals who live in a similar environment as mice do not have a UV vision or dorsoventral functional division as in the mouse retina. We clarified the point in the **revised Discussion section (lines 449-455)**.

Can the authors make any predictions about chromatic contrast, and how their measurements may relate to this aspect of vision?

Following the methods in Qiu et al., (2021), we analyzed the chromatic contrast of our image datasets (**new Fig 4**). Specifically, we first computed the root mean square (RMS) contrast for each channel of each acquired image:

C*(x,y) = σ*(x,y) / μ*(x,y),	(Eq.4 in the revised manuscript)

where μ*(x,y) and σ*(x,y) are the mean and standard deviation of the circular image patch (radius, 30 pixels) centered at location (x,y), respectively; and the asterisk “*” is either “UV” or “green” indicating the channel identity. We then took a difference of the RMS contrasts between the two channels to assess the chromatic contrast of individual images:

C(x,y) = C^UV^(x,y) - C^green^(x,y),	(Eq.5 in the revised manuscript)

and computed the probability distributions between the upper and lower field images. For details, see the **revised Methods section (lines 172-182)**.

Consistent with Qiu et al. (2021), we found that the RMS contrast was higher in UV than in Green channels, especially for the upper field images (Fig 4A,B). The upper visual field images then had an asymmetric chromatic contrast distribution (Fig 4C), where pixels with higher contrast in UV than in Green were more abundant than those with higher contrast in Green than in UV (Fig 4E,F). In contrast, the chromatic contrast distribution was rather symmetric for the lower visual field images (Fig 4D), and overall wider than that for the upper visual field images (Fig 4E,F). This also agrees with what Qiu et al (2021) observed in the “high” intensity images (see Fig 3F in Qiu et al 2021). Qiu et al (2021), however, drew a different conclusion as they identified a different result in the “low/medium” intensity images (as well as in their modelling analysis).

Here we identified in our image datasets that UV-green chromatic information surely exists across the visual field (see examples in Fig 2 and S2 Fig). We cannot then make a clear prediction on the chromatic circuitry in the mouse retina. In principle, mice can retrieve UV-green chromatic information across the visual field, given that 1) genuine S-cones and rods are distributed rather uniformly across the mouse retina (Jeon et al., 1998); 2) rods have similar absorption spectra to M-cones (peak sensitivity at 498 and 508 nm, respectively; revised Fig 1B; Bridges, 1959; Jacobs et al., 1991); and 3) rods can escape from saturation even under photopic conditions (Tikidji-Hamburyan, et al., 2017). Why does the mouse retina have chromatic vision only on the ventral side (upper visual field; Joesch and Meister, 2016; Szatko et al., 2020; Nadal-Nicolás et al., 2020)? It is suggestive that chromatic filters emerged in convolutional auto-encoder models only when the upper field images are trained but not the lower field images (see Fig 6 in Qiu et al 2021). The outcome, however, depends on the training datasets. Many natural objects on the ground do have a good UV-green contrast, such as gloss of leaves and water (see examples in Fig. 2 of our paper), and these objects may not be included in the datasets used in the modelling analysis by Qiu et al. (2021). Moreover, we do not know yet how much the mouse eye optics create image distortion (and how the mouse visual system compensates for the distortion). Thus, one has to be careful in interpreting the modelling results. Future studies should use larger datasets acquired under more diverse conditions, especially because the rod system is involved not just in the color vision but also in the scotopic vision. We described this new analysis results in the **revised Results section (lines 323-355)**.

Relatedly, what about rod photoreceptors? There is increasing evidence that rods are also active in brighter light, and at least some discussion of this fact is needed in this manuscript.

The photon flux on individual photoreceptors can be estimated by the following equation:

Φ * A_pupil_ / A_retina_ * T * A_photoreceptor_,

where Φ is the illuminance of the environment; A_pupil_ = π (d_pupil_/2)^2^ is the pupil area (with diameter d_pupil_); A_retina_ = 4 π (d_eye_/2)^2^ / 2 is the total area of the retina (covering a half of the eye internally with diameter d_eye_); T is the transmittance of the eye opics; and A_photoreceptor_ is the light collection area of a photoreceptor. In this study, we took images under ample light during the day. Although we did not measure the exact illuminance, we thus expect that the lighting condition was on the order of 10^3^-10^5^ lux (Φ = 10^7^-10^9^ photons/μm^2^/s). Assuming d_pupil_=0.5 mm, d_eye_=4 mm, T=0.5, and A_photoreceptor_=0.5 μm^2^, we then have 10^4^-10^6^ photons/photoreceptor/s. As the reviewer pointed out, a recent study suggests that the mouse rods may escape from saturation even at 10^7^ R*/ rod/s (Tikidji-Hamburyan, et al., 2017). Thus, we cannot exclude a possible contribution of rods in processing the natural images we acquired. This point is clarified in the **revised Methods section (lines 110-123)**

Rods have similar absorption spectra to M-opsin expressing cones () and are distributed rather uniformly in the mouse retina, occupying ~97% of all photoreceptors (Jeon et al., 1998). Thus, sampling of the green channel by the rods should be dense and uniform across the mouse visual field, despite distinct image statistics between the upper and lower visual field images. This suggests that the distribution of the mouse rods is not adapted to the image statistics in the photopic condition *per se*, likely due to a stronger selective pressure to work in the scotopic condition. We clarified this point in the **revised Results section** (**lines 345-355**).
