# Supplementary material for: Natural image statistics for mouse vision
Source: PLoS One. 2022 Jan 20;17(1):e0262763. doi: 10.1371/journal.pone.0262763 (PMC8775586; doi:10.1371/journal.pone.0262763)
Supplement: Attachment — Submitted filename: Response_to_reviewers.docx [file pone.0262763.s006.docx]

We thank the reviewer #1 for his/her careful reading of our manuscript and constructive comments (in blue). Below are our point-by-point replies (in black) to address all the remaining concerns.

Reviewer #1: The authors have made an extensive effort to address my concerns. The manuscript is improved a lot. I am afraid I remain unclear on the following points:

1) The title “Natural Image Statistics for Mouse Vision” does not specifically fit for what is described in the paper. The natural image statistics include many aspects such as first-order, second-order and higher-order statistics. In this study, only the contrast was massively analysed, while many other aspects remain untouched.

The main focus of this study is not the natural images for mouse vision *per se*. Instead, we are interested in the effects of the natural image statistics on the mouse vision from an optimality viewpoint. In particular, we asked why cone photoreceptors in the mouse retina have a distribution bias in the dorsoventral axis, and demonstrated that it cannot be explained by the natural image statistics for mouse vision from the information theoretic perspective of the efficient coding principle. We clarified the point in the revised manuscript **(lines 10-11, 47-48, 215-217)**.

In the original manuscript, we focused on the first- and second-order statistics because 1) it has been shown that the retina itself is sensitive up to the second-order image statistics, but not to higher-order ones (Bonin et al 2006; Tkačik et al 2014); and 2) there is no straightforward prediction that can be derived from the higher-order image statistics on the mouse retinal organization. We clarified this point in the revised manuscript (**line 145**). Nonetheless, we also included the higher-order statistics of our image datasets (kurtosis, skewness, and entropy of local image patches; **lines 183-185** and **S4 Fig** in the revised manuscript) as a reference for future studies. We thus believe that the title “natural image statistics for mouse vision” fits well to the scope of this study.

In addition, the camera system only respected the spectra of the mouse retina.

Spectral bands outside the spectral range of the mouse retina are irrelevant to the objective of this study, i.e., to examine if the natural image statistics for mouse vision can explain the distribution bias of the cone photoreceptors in the mouse retina. Natural image statistics for human vision have been extensively studied in previous studies (e.g., Hyvärinen et al., 2009).

But the visual angle may only represent the FOV of no more than 5 RGCs. Although the authors expressed that “… natural image statistics are scale invariant…” (line 164-165) and I also agree with this in terms of statistics, this small FOV (< 11.3 degrees) cannot represent mouse vision (180 degrees).

It is true that none of our images cover the entire mouse visual field. We pointed this out as a caveat in the revised Discussion (**line 428**). However, our image datasets well represent the upper and lower visual field at the level of the image statistics, which is the key feature needed in this study. More importantly, spatial correlation/power spectrum analyses (Figs. 5 and 6) require images without any spatial distortion (and vignetting). A small field of view (FOV) is thus preferable than a large FOV in this regard.

2) The authors emphasized the advantages of this camera system by 1) smaller FOV for low distortion, 2) high-performance sensor with higher dynamic range and higher resolution, and 3) fewer optical elements ensure transmission of near UV (line 72-81). Nevertheless, this camera system was designed for mouse vision. So, the small FOV probably has the bad impression compared to real mouse vision (180 degrees). Meanwhile, Increasing the FOV can spontaneously solve the problem of focal depth.

Our camera system was designed for analyzing the UV-green image statistics to test how optimal the mouse vision/retina is to the image statistics. Capturing the entire monocular (180 degrees) or binocular visual fields for recapitulating “real” mouse vision is thus beyond the scope of this study.

Also, the mouse retina has low resolution output depends on the density of RGCs (3 to 13 degrees). Thus, the described advantages of FOV (50mm lens) and high resolution (camera sensor) mostly refer to the human eye’s perspective.

Here we focused on the sampling bias of the mouse retina at the level of photoreceptors (input neurons), not at the level of ganglion cells (output neurons). Given the average cone density of 12,400 cells/mm^2^ (Jeon et al., 1998) and the eye diameter of 4 mm, the sampling resolution (or the “pixel size”) of the mouse visual system is on the order of 0.25 degrees (= 180/( sqrt(12400) * 4/2*pi ) for photopic vision), and can go as high as 0.05 degrees if rod photoreceptors are also involved (average density, 437,000 cells/mm^2^ (Jeon et al., 1998); or average diameter of 1.4 μm (Carter-Dawson and LaVail, 1979)). This is the upper limit of the spatial resolution that the mouse visual system can exploit to a first approximation. As the reviewer pointed out, this is much higher than the spatial resolution of the retinal output level (RGC receptive fields: 3-13 deg) or the visual acuity of mice (0.5-1 cycles/deg); however, it is a different issue how such information is processed within the retina and the downstream visual pathway. Given that the pixel size of mouse vision is on the order of 0.1 - 0.01 degrees, we argue that the spatial resolution of our image datasets (0.006 degrees/pixel) is relevant to mouse vision and appropriate to examine the relationship between the image statistics and the mouse retinal organization. We clarified these points in the revised manuscript (**lines 124-128**).

In addition, the optical elements are not fewer compared to the other system cited in the paper. There are already 6 lenses in 5 groups from the Nikon commercial camera lens and two filters in each chromatic channel.

We agreed with the reviewer on this point and removed the corresponding part from the manuscript (**lines 81**). We, however, would like to reiterate that the Nikon lens is designed to minimize lens distortion (yet has a good near-UV transmission). Thus, it has an advantage over a fisheye lens (Qui et al., 2021) or panoramic system (Differt & Möller, 2015, 2016) for image statistics analysis, in particular, spatial autocorrelation / power spectrum (Figs. 5 and 6).

3) As the authors did not perform the intensity calibration of the camera system, I would like to suggest doing baseline/background subtraction before the normalization. The high-performance sensor has a big dynamic range. However, it still has its minimum detectable signal, like other sensors in the market. This effect can be nicely explained from revised Fig. 1A inset. The line was not able to pass through the origin of the coordinate system and the y-intercept is the effective minimum sensitivity of the camera sensor. When reflecting in the images, it was exactly shown by the UV-green pixel intensity distributions (S2 Fig) where one cannot find any values at 0, instead the darkest points were accumulated at the values close to zero. In fact, those data dots contain no real luminance information, meaning underexposure, which should be excluded in the statistic calculations. In this case, contrast should be calculated based on images with baseline/background subtraction.

Thanks for the suggestion. This is exactly what we did from the beginning, as described in the methods section (formerly called “Normalized light intensity”, lines 143-149 in the first manuscript / lines 153-158 in the first revision; now called “Light intensity normalization”, **lines 159-164** in the second revision).

4) In the results part, the authors expressed no public database of natural scenes covering UV images (line 205-207). Here I would like to suggest the authors check the three publications below:

-Differt & Möller (2015) Insect models of illumination-invariant skyline extraction from UV and green channels. J. Theor. Biol. 380, 444–462

-Differt & Möller (2016) Spectral skyline separation: extended landmark databases and panoramic imaging. Sensors 16, 1614

-Qiu et al (2021) Natural environment statistics in the upper and lower visual field are reflected in mouse retinal specializations. Curr Biol. 2021 Aug 9;31(15):3233-3247.e6.

Thank you very much for the suggestions. We have cited these publications and revised the manuscript (**lines 73-74, 215**) to clarify that our datasets are complementary to the existing ones. Namely, our image datasets cover upper and lower visual fields at a high resolution with minimal lens distortion. To our knowledge, none of the images in these publications were calibrated or corrected for vignetting or spatial distortion.

5) Again, there is a mistake of the hardware information that the short pass filter (Edmund Optics 84-708) should cut at 550nm, not 500nm.

Nevertheless, the method is appropriately described here, and the paper as a whole represents an important contribution to the public database for nature images of UV.

We sincerely apologize for the typo. Here we used a 550 nm short-pass filter to make sure to cut the small IR components that Hoya U-340 filter can transmit. We have corrected the text in the revised manuscript (**line 62**).
